# Bibliometric Analysis of Global Research on Tumor Dormancy

**DOI:** 10.3390/cancers15123230

**Published:** 2023-06-18

**Authors:** Yuzhe Zhang, Lirong Yan, Zhongqing Wang, Fang Li, Jinqi Lv, Jiaqing Liu, Xuqin Liu, Li Bao, Ye Zhang

**Affiliations:** 1 The First Laboratory of Cancer Institute, The First Hospital of China Medical University, Shenyang 110001, China; 2 Department of Information Center, The First Hospital of China Medical University, Shenyang 110001, China

**Keywords:** tumor dormancy, bibliometric, immunotherapeutic treatments, microenvironment, mechanism

## Abstract

**Simple Summary:**

Tumor dormancy continues to be a research hotspot with numerous pressing problems that need to be solved. The goal of this study is to perform a bibliometric analysis of pertinent articles published in the twenty-first century. We concentrate on significant keywords, nations, authors, affiliations, journals, and literature in the field of tumor dormancy, which will help researchers to review the results that have been achieved and better understand the directions of future research. We can comprehend the evolution of the field more rapidly thanks to the abundance of visual information. We can better grasp some significant discoveries and scientific advances by analyzing some key works in the subject. We can also more immediately spot pressing concerns and issues. We believe that research on tumor dormancy has been a popular subject. Future research areas that are anticipated to be most popular include the investigation of the tumor dormancy microenvironment and immunotherapeutic therapies for tumor dormancy.

**Abstract:**

Tumor dormancy continues to be a research hotspot with numerous pressing problems that need to be solved. The goal of this study is to perform a bibliometric analysis of pertinent articles published in the twenty-first century. We concentrate on significant keywords, nations, authors, affiliations, journals, and literature in the field of tumor dormancy, which will help researchers to review the results that have been achieved and better understand the directions of future research. We retrieved research articles on tumor dormancy from the Web of Science Core Collection. This study made use of the visualization tools VOSviewer, CiteSpace, and Scimago Graphica, as visualization helps us to uncover the intrinsic connections between information. Research on tumor dormancy has been growing in the 21st century, especially from 2015 to the present. The United States is a leader in many aspects of this research area, such as in the number of publications, the number of partners, the most productive institutions, and the authors working in this field. Harvard University is the institution with the highest number of publications, and Aguirre-Ghiso, Julio A. is the author with the highest number of publications and citations. The keywords that emerged after 2017 were “early dissemination”, “inhibition”, “mechanism”, “bone metastasis”, and “promotion”. We believe that research on tumor dormancy mechanisms and therapy has been, and will continue to be, a major area of interest. The exploration of the tumor dormancy microenvironment and immunotherapeutic treatments for tumor dormancy is likely to represent the most popular future research topics.

## 1. Introduction

In recent years, tumor dormancy has been recognized by scientists as an important stage in tumor development. It refers to the presence of tumor cells when tumor progression is not clinically evident [1]. In clinical practice, tumor dormancy refers to the state of the tumor before the occurrence of secondary site metastasis following the treatment of the primary tumor [2]. This phenomenon is an important reason why malignant tumors persist and are difficult to cure. It is not clear when these dormant cells cause tumor recurrence [3], nor which cells suddenly return to a proliferative state and through which specific mechanism this occurs [4].

To better address the difficulties in tumor dormancy research, it is necessary to understand most of the basic knowledge and to build a multidimensional research network using longitudinal and global perspectives to summarize past results and indicate possible future research directions. Bibliometrics first appeared in the early 20th century and became an independent discipline in 1969, being widely used for documentary analysis [5]. We can analyze existing publications to better understand the authors, their institutions, and the status of a journal’s research. More importantly, this can provide us with a greater awareness of the latest research hotspots and emerging themes for future research.

## 2. Materials and Methods

The core collection of Web of Science, a feature-rich database of high-quality digital literature resources with sufficient bibliometric indicators to facilitate our analysis [6], was selected as the most suitable database for bibliometric analysis in this study [7].

**Figure 1:**
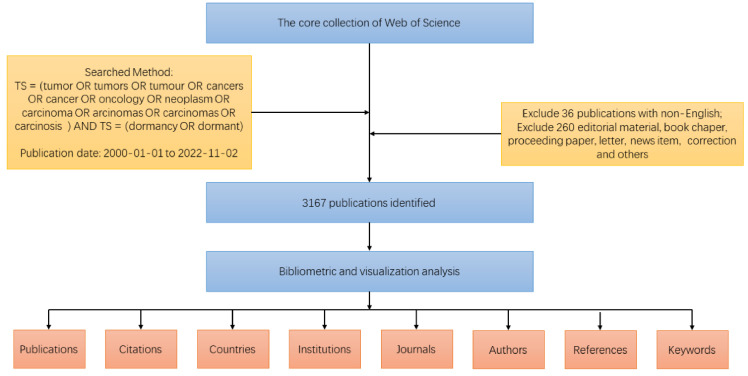
Diagram showing the process for finding, filtering, and evaluating articles on tumor dormancy.

The search strategy is shown in Figure 1. On 3 November 2022, we performed a literature search using the Web of Science Core Collection (WoSCC) database [8]. We used the following search strategy: TS = (tumor OR tumors OR tumour OR cancers OR cancer OR oncology OR neoplasm OR carcinoma OR carcinomas OR carcinosis) AND TS = (dormancy OR dormant). We excluded non-English articles (*n* = 36), editorial materials, book chapters, proceedings papers, letters, news items, corrections, and irrelevant articles (*n* = 260), resulting in a final selection of 3167 articles. The complete record of each publication, including the title, year of publication, author name, nationality, affiliation, journal name, keywords, and abstract, was downloaded from the Web of Science database and imported into Microsoft Excel 2016 [9]. We used an online website (https://charticulator.com, accessed on 1 May 2023) for our analysis.

This study made use of the visualization tools VOSviewer (v.1.6.18), CiteSpace (6.1.R6), and Scimago Graphica (v.1.0.26). VOSviewer is a classic bibliometric analysis software that uses a probabilistic-based approach to data normalization to produce aesthetically pleasing images [10]. CiteSpace is a bibliometric analysis and visualization tool that uses institutions, keywords, and other data to identify the dynamics of a certain scientific area. The global distribution of national publications is mapped using Scimago Graphica [11].

In order to analyze the genes and proteins referenced in the gathered keywords, we set a minimum threshold of five keyword occurrences. A biomedical named entity recognizer, which is used to tag genes, proteins, and biological entities, was used to recognize and tag the genes and proteins in the keywords [12]. The Entrez Global Query CrossDatabase Search System (https://www.ncbi.nlm.nih.gov/Web/Search/entrezfs.html, (accessed on 1 May 2023)) then normalized the genes and proteins present, yielding 46 genes [13]. The Kyoto Encyclopedia of Genes and Genomes (KEGG) pathway study by Gene Ontology (GO), Kyoto Encyclopedia of Genes, and other methods followed the enrichment analysis of genes related to tumor dormancy. The data for the protein–protein interaction network were obtained from the STRING database [14] (http://www.string-db.org/ (accessed on 1 May 2023)) and were then loaded into the Cytoscape 3.9.1 software for analysis using the Cytohubba plugin [15].

## 3. Results

### 3.1. Posting Volume Worldwide and Country Cooperation

**Figure 2:**
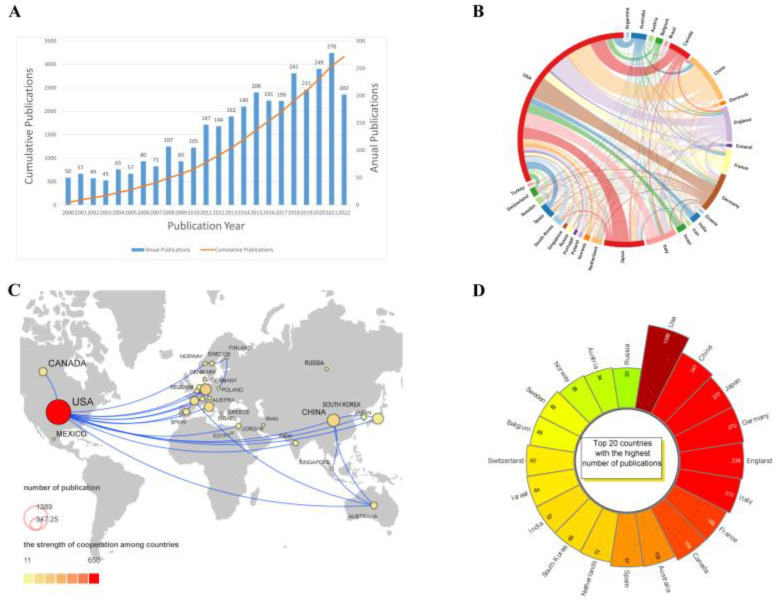
(**A**) Global publication trends of articles related to dormant tumors. (**B**) Map of the world showing the number of publications by nation. The number of publications is represented by the size of the circles, and the level of collaboration between the two nations is indicated by the thickness of the lines. (**C**) A network diagram showing international cooperation. (**D**) Top 20 countries with the highest number of articles.

From 2000 to 2022, a total of 3167 articles were published that met the search criteria. A quantitative analysis of published articles about tumor dormancy is shown in Figure 2A. We can see that the number of articles published annually from 2000 to 2007 was always between 50 and 100 articles. From 2009 to 2015, the number of publications generally maintained a steady growth rate. From 2015 to the present, there has been a boom period, with 1768 articles being published during this period, representing 55.82% of the total number of articles published in the 21st century.

Table 1 lists the top ten countries that contributed to publications in this field of study, with the United States publishing the highest number of articles (1389, 43.85%). Publications from the United States were also cited the most frequently (82,193 citations). Articles from Australia had the highest average citation frequency (72.25 citations), indicating the high quality of Australian publications. In Figure 2B,C, the national collaboration networks are displayed. The top twenty nations with the most articles posted are displayed in Figure 2D. 

### 3.2. Analysis of Institutions

**Figure 3:**
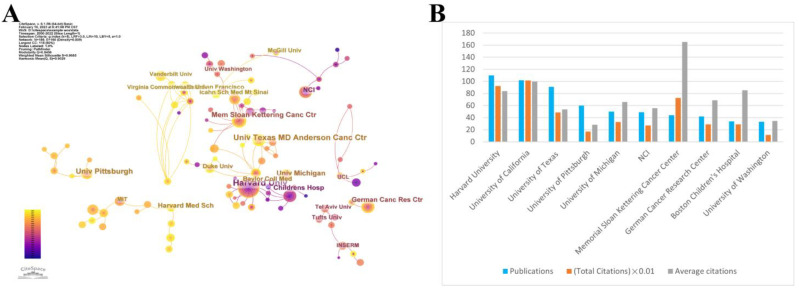
(**A**) Top 10 most productive institutions and institutional alliances. (**B**) The top 10 institutions were ranked based on the number of publications, total citations, and average citations.

Figure 3A depicts the collaborative networks of research institutions. These institutions make up collaborative network clusters denoted by various colors. According to the number of publications published, Figure 3B shows the top 10 organizations, Harvard University (110) is the most productive institution, while the Memorial Sloan Kettering Cancer Center has the highest average number of citations with 165.25, meaning that this institution produces a large amount of impactful research.

### 3.3. Author Collaborations

**Figure 4:**
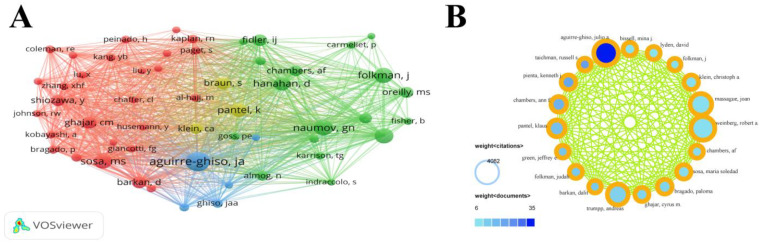
(**A**) Graphics for the study of authors’ co-citations. The size of the points represents the total link strength. The established working relationship between the two authors is represented by the line connecting the two points. The degree of collaboration between the two authors is shown by the line’s thickness. (**B**) The number of publications and citations of 19 authors.

A relevant analysis of authors helps with identifying the core authors and major collaborations in the field. The top ten authors were selected based on the number of citations. Their affiliations, total link strengths, number of publications, and number of citations are shown in Table 2. We only included authors with more than five publications in the field. The author with the highest number of citations was Aguirre-Ghiso, Julio A. (4082). We filtered the authors with more than 100 citations, and their co-citation analysis is presented in Figure 4A with four different author clusters. In addition, in Figure 4B, we filtered the 19 authors by the number of articles > 5 and a number of citations > 1000.

### 3.4. Examination of Publications and Journals

The top 10 journals with the highest number of publications and their 2021 JCR divisions are shown in Table 3, and the combined volume of publications in these 10 journals represents 20.92% of the tumor dormancy-related literature over these two decades. 

Among the top 10 journals, Cancers, International Journal of Molecular Sciences, and Scientific Reports have average years of publication of 2020.66, 2019.25, and 2018.25, respectively, which indicates that these three journals have published a large number of relevant articles in recent years. 

**Figure 5:**
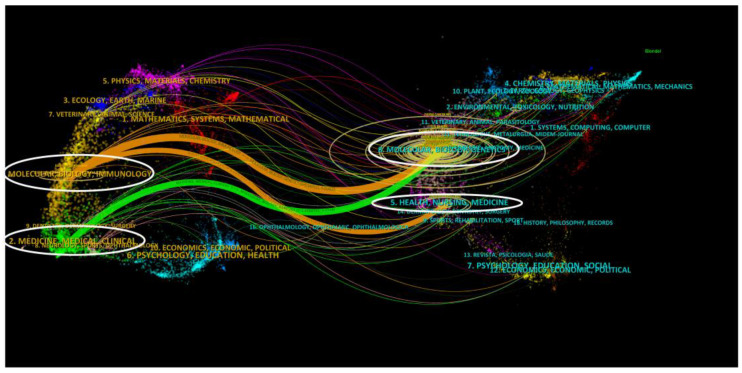
The dual-map overlay of journals of tumor dormancy research. The left circles were targeted literature, while the right circles were source literature.

Dual-map overlay analysis can help us to understand the flow of knowledge between publications and the changing trends within disciplines. The left side of Figure 5 represents the distribution of journals in which the cited literature is located, while the right side shows the distribution of journals in which the cited literature is located. We have found that research in the field of tumor dormancy is mainly related to immunological, biological, and molecular aspects and is frequently cited by researchers in the molecular, genetic, and biological fields.

### 3.5. Analysis of Co-Cited References

**Figure 6:**
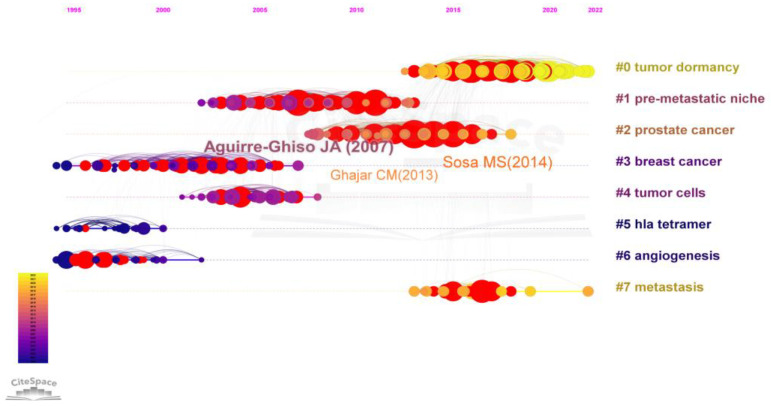
Timeline view of co-cited references. A cluster is represented by each horizontal line; the greater the horizontal line and the largest cluster is #0. The linkages show co-cited associations, and the node size represents co-citation frequencies. The node and line colors indicate distinct years, and the nodes are at their initial co-cited year. Solid lines indicate hot clusters for particular years.

It is important to group-specialized materials in particular domains and to examine the historical moments at which particular items first emerged. We analyzed the co-citations of the cited references, aiming to uncover as much important information as possible. The 10 documents with the highest number of co-citations are displayed in Table 4. Notably, three of the top ten articles are by Aguirre-Ghiso, Julio A., who also has the highest total number of citations, indicating that Aguirre-Ghiso, Julio A. has made outstanding contributions to the field of tumor dormancy. The reference co-citation network diagram in Figure 6 shows how all nodes can be divided into eight primary clusters. The most often researched term is the label of each cluster. The most prevalent clusters are clusters #0 (tumor dormant) and #1 (pre-metastatic niche).

### 3.6. Analysis of Keywords

**Figure 7:**
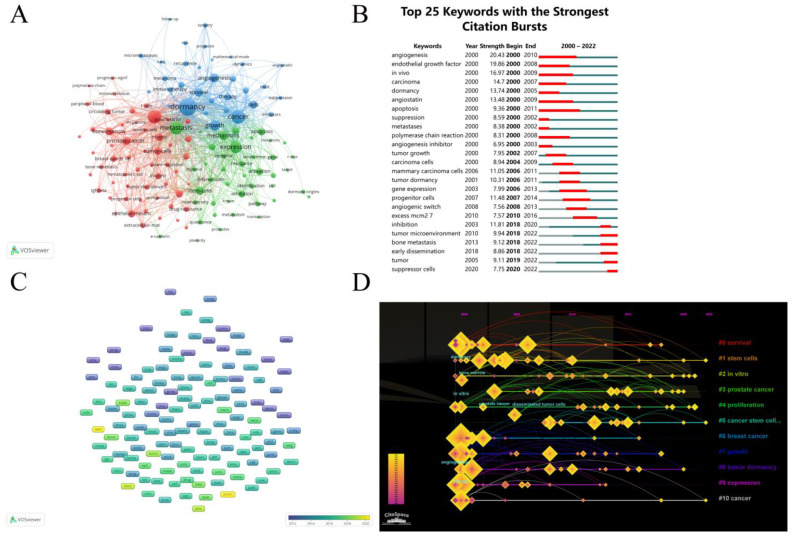
(**A**) Mapping the co-occurrence of important keywords. There are 129 keywords, and the minimum number of occurrences of each keyword is 30. (**B**) The top 25 keywords and their intensity, with red bars showing the year of their outbreak. (**C**) Keyword distribution is based on the average publishing year. (**D**) The network of keywords’ co-occurrences is represented visually. Based on color, keywords are classified into eleven groups. High-frequency keywords are represented by large nodes.

A keyword analysis is useful for us to establish a framework for research on tumor dormancy. As shown in Figure 7A, the minimum number of keyword occurrences was set to 30, and 129 keywords were obtained, showing that the topic terms were divided into three clusters. In Figure 7C, the keywords were colored according to the average appearing year (AAY) of publications, with yellow-coded keywords indicating the most recent publications. The recently appearing keywords are “promoted” (2020.12), “early dissemination (2019.70), and exosomes (2018.97). The top 25 keywords with the strongest citation burst are listed in Figure 7B. Figure 7D displays a timeline view of the keywords. A cluster is represented by a horizontal line, with #0 (survival) being the biggest cluster.

Most of these keywords indicate key molecules and diseases, as shown in Table 5, which can help us to clarify some of the major research directions in the field of tumor dormancy. The most frequently addressed diseases in this field include breast cancer, prostate cancer, and colorectal cancer. Dormancy, metastasis, and angiogenesis are the top three physiological or pathological states associated with tumor dormancy research. 

### 3.7. Hub Genes and Pathways Analysis

**Figure 8:**
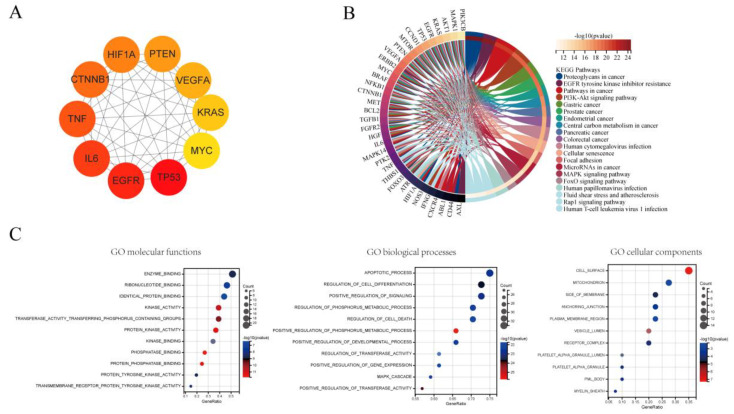
Analysis of genes related to tumor dormancy from 2000 to 2022. (**A**) Ten hub genes. (**B**) KEGG pathway analysis. (**C**) Results of GO analysis of biological processes, molecular functions, and cellular components.

A total of 46 genes were found to be essential for tumor dormancy. The 10 most important genes, according to the PPI network, were TP53, EGFR, IL6, TNF, CTNNB1, HIF1A, PTEN, VEGFA, KRAS, and MYC (Figure 8A). Proteoglycans in cancer, EGFR tyrosine kinase inhibitor resistance, and the cancer pathway were found to be the top three KEGG indicators (Figure 8B). These genes play crucial roles in binding enzymes, ribonucleotides, identical proteins, and other molecules. The apoptotic process and cell differentiation regulation are the two primary biological processes. Additionally, these genes are linked to biological elements, including the cell surface, mitochondrion, membrane side, etc. The primary biological processes include those that control cell development, signaling, and apoptosis (Figure 8C).

## 4. Discussion

Tumor dormancy was defined by Willis in the 1940s [16], but it was not until the 21st century that researchers decided to focus more on this phenomenon [17]. Trends in annual and total publications in the field of tumor dormancy show that more than half of the relevant studies were published between 2015 and 2022, indicating that increasing numbers of researchers are becoming enthusiastic about tumor dormancy research. Furthermore, relevant research is likely to remain a focus in the future as there are many outstanding questions in the field of tumor dormancy regarding the detailed mechanisms of key molecules, the specific roles of relevant signaling pathways [18], and the development of specific targeted drugs [19].

In terms of countries, the United States leads in this area of research, not only publishing the most relevant literature (1389, 43.85%) but also dominating in terms of national collaborations. In turn, as many as nine of the ten most productive institutions belong to the United States, and these institutions are extensively involved in close inter-institutional collaboration. This suggests that advanced research institutions may be important to a country’s academic excellence. Based on the quantity and quality of the relevant literature published in journals, we have found core journals in the field of tumor dormancy. Nine of these journals are from JCR regions I and II and have high academic value, so we can follow these journals to understand the academic dynamics of the field and consider them when submitting relevant papers. Cancer Research is firmly in first place in terms of the total number of publications and the total number of citations, while Clinical Cancer Research has the highest average number of citations. Cancers ranked first in the average years of publication, indicating that it has focused more on tumor dormancy research in recent years.

Three of the top ten most highly co-citation of cited references were from Aguirre-Ghiso, Julio A., confirming Aguirre-Ghiso, Julio A.’s outstanding contribution to the field of tumor dormancy. Within these three papers, a review was published in 2007 in which he summarized the research evidence supporting tumor dormancy and the concepts of angiogenic dormancy, cellular dormancy, and immune-mediated dormancy that are widely accepted by scientists [20]. To date, dormancy at the cellular level has remained a research area of great attention [21]. Tumor dormancy at the cellular level is usually associated with DTCs (lacking markers of proliferation and apoptosis) in the quiescent phase; therefore, factors associated with proliferation reflect, to some extent, the state of tumor cells [22].

To observe how cells transition between quiescent and proliferative stages, we can track gene expression [23]. The expression of Ki67, M30, and the more well-known terminal deoxynucleotidyl transferase dUTP nick end labeling (TUNEL) assay can also help us to determine whether tumor cells are in a dormant state from the perspective of apoptosis, in addition to the pERK/p38 ratio, which directly reflects the status of DTCs. In recent years, it has also been discovered that cancer cells in a dormant state exhibit higher levels of P38 and Notch. Additionally, autophagy [24] may support DTC adaptability and encourage the survival of DTCs [25], where the EGFR blocks the AKT signaling pathway and prevents the formation of cyclin D1 [26]. Autophagy is in some way intimately related to tumor dormancy, according to studies on ARHI in ovarian cancer [27]. The expression levels of autophagy-related genes 4 and 8 (ATG4 [28] and ATG8) support the hypothesis that autophagy and dormancy are somehow connected.

Meanwhile, following the publication of this influential review article, the number of publications in the field of tumor dormancy surged in 2008. A review published in 1995 suggested that tumor dormancy may represent a balance between tumor cell proliferation and apoptosis, clarifying the role of angiogenesis in tumor dormancy. Later, in 1998, researchers identified dormancy in individual cells, identifying potential targets for therapeutic strategies. A review published in 2014 concluded that disseminated tumor cells (DTCs) have some metastatic growth capacity [29]. This explains why metastases with a different nature to primary lesions are generated and why we have difficulty overcoming tumor metastasis despite therapeutic agents targeting the primary lesion. The analyses of the co-citation of cited references helped us to understand the milestone events in the field of tumor dormancy.

We used symbiotic analysis to identify the areas of interest and hot topics in the field of tumor dormancy and expected that this could guide researchers to conduct more in-depth research. We focused on keywords that appeared more than or equal to 30 times in the field and performed a cluster analysis using VOSviewer; these keywords were divided into three clusters, representing different research directions and hot topics (Figure 7A).

The prominent keywords of the largest red cluster include “breast cancer”, “bone-marrow”, and “prostate cancer”, among others. The cluster focuses on the association between tumor dormancy and bone marrow. The association between bone marrow and dormant tumor cells has been of interest to researchers since the beginning of this century [30], thanks to the pattern of cancer metastasis; for example, breast cancer often metastasizes to bone [31], and prostate cancer preferentially spreads to bone [32]. Among the top ten documents with the highest number of co-citations listed in this article, one document published in 2002 suggested that it is important to understand the factors that influence the specific metastatic growth of different types of tumors in different organs. 

Additionally, in these ten highly cited papers, two studies from 2013 uncovered key factors in the bone marrow microenvironment that influence breast cancer cell dormancy (TGFβ2 signaling in the bone marrow microenvironment can induce dormancy in DTCs [26], and factors deposited in endothelial cells and in their surroundings may be involved in tumor dormancy [33]). This corroborates our hints about research hotspots from the keyword analysis. There is growing evidence that disseminated tumor cells from epithelial-derived tumors such as breast, prostate, and colorectal cancers are often present in the bone marrow [34]. Recent studies suggest that cancer-derived exosomes have been found to be involved in remodeling the tumor microenvironment, promoting angiogenesis, and modulating the immune system and that some exosomes can secrete epithelial cell adhesion molecules (EpCAM) [35].

On the other hand, in addition to the widely used transgenic mice and two-dimensional cellular models, three-dimensional bioengineering models inspired by tissue engineering strategies [1] are gaining increasing attention [36]. These models [37] have achieved significant breakthroughs in mimicking the tumor microenvironment, understanding the regulation of tumor dormancy by the tumor microenvironment, and exploring the interactions between tumor cells and the microenvironment [38]. In addition, when immunotherapy is of great interest [39], co-culture systems containing other cells, such as immune cells designed using 3D models, may help us improve our success rate regarding drug screening and validation [40]. Therefore, in the future, research will still focus on several cancer tissues, as mentioned above, and studies on tumor dormant tumor microenvironments will remain prevalent [41].

The green cluster focused on studies about the mechanisms of tumor dormancy. The mechanisms of tumor dormancy can be simply divided into the following two types: intracellular mechanisms represented by altered signaling pathways, including the p38 MAPK signaling pathway [42], TGFβ signaling [43], Wnt signaling axis [44], Notch2 pathway [45], etc., and extracellular mechanisms related to the tumor microenvironment, angiogenesis, and immunity. Although scientists have been exploring this field for decades, the mechanisms of tumor dormancy are complex and have only been partially elucidated [46]. Combined with the aforementioned keywords compiled here, the top ten most frequent molecules and diseases suggest that researchers have never stopped exploring the main factors leading to tumor dormancy [47]. This is extremely important for us to identify new targets and develop more effective targeted therapies [48]. This will remain a topic of significant research value in the future [49].

The blue cluster focuses on the treatment of tumor dormancy. In cancer patients and cancer survivors, the existence of dormant tumor cells is the cause of tumor recurrence [50]. Since dormant tumor cells are inactive, neither chemotherapy nor radiation therapy will affect them. In contrast, chimeric antigen receptor (CAR) therapy, immune checkpoint inhibitors, and pericyte therapy using lymphocytes offer new hope for tumor treatment [51]. The role of T cells in tumor dormancy is undisputed, and the helper T cell (Th) subtypes Th1 [52] and Th2 [53] have also been found to secrete cytokines involved in tumor growth and metastasis. Recently, activated hepatic stellate cells (HSCs) were found to secrete CXCL12 while suppressing the proliferation of co-cultured NK cells, thereby promoting the awakening of dormant DTCs, and causing metastasis [54]. This suggests that therapy could be designed to maintain the normalization of NK cells. Research on immunotherapy is still being conducted extensively [55].

It is important to note that our understanding of tumor dormancy is still only partial, and research targeting tumor dormancy mechanisms and therapeutic approaches has never been compartmentalized. Autophagy-related genes [56], inflammatory factors [31], factors regulating angiogenesis, and immune cells [57] have all been found to be involved in the process of tumor dormancy [58], and we can use this to develop specific therapies once the mechanisms of dormancy occurrence are clarified [18].

As a bibliometric analysis, this study has two limitations. All data were retrieved and downloaded from the Web of Science (core collection). Although this database is considered the most suitable database for bibliometric analysis, papers not included in this database can be missed. Additionally, because we restricted the study to English literature, other significant studies in other languages might have been missed [59].

## 5. Conclusions

Based on a bibliometric analysis of the tumor dormancy field, detailed information on a large number of publications can be more intuitively understood through visual or cluster analysis. Research on tumor dormancy has been growing over the past twenty-two years, especially from 2015 to the present day. The United States is the leader in almost all aspects of research, such as the volume of publications, productive institutions, and authors. Harvard University is the institution with the highest number of publications, and Aguirre-Ghiso, Julio A. is the author with the highest number of publications and citations. More importantly, research on the mechanisms and treatments of tumor dormancy has been and will continue to be, an extremely hot topic, considering that dormant tumor microenvironments and immunotherapy are probably the most critical areas of research.

## Figures and Tables

**Table 1:** The top 10 productive countries.

Rank	Country	Documents	Citations	Average Citations
1	USA	1389	82,193	59.17
2	China	347	8814	25.40
3	Japan	272	8954	32.92
4	Germany	270	19,499	72.22
5	England	238	13,470	56.60
6	Italy	210	9530	45.38
7	France	158	6907	43.72
8	Canada	154	8238	53.49
9	Australia	105	7586	72.25
10	Spain	97	5427	55.95

**Table 2:** The top 10 authors with the highest number of citations.

Rank	Author	Affiliation	Total Link Strength	Documents	Citations
1	Aguirre-Ghiso, Julio A.	Albert Einstein College of Medicine	2165	35	4082
2	Weinberg, Robert A.	Whitehead Institute	187	7	3862
3	Massague, Joan	Memorial Sloan Kettering Cancer Center	233	7	3153
4	Trumpp, Andreas	German Cancer Research Center	48	12	2940
5	Pantel, Klaus	University Medical Center Hamburg-Eppendorf	403	16	1922
6	Chambers, Ann F.	University of Western Ontario	722	19	1653
7	Bragado, Paloma	Hospital Clinico San Carlos	795	11	1476
8	Pienta, Kenneth J.	Johns Hopkins Medicine	657	20	1450
9	Klein, Christoph	University of Regensburg	306	7	1422
10	Sosa, Maria Soledad	Icahn School of Medicine at Mount Sinai	685	8	1400

**Table 3:** The top 10 journals with the most documents.

Rank	Journal	JCR (2021)	Documents	Citations	Average Citations	Average Publication Year
1	Cancer Research	Q1	221	8191	37.06	2013.33
2	Cancers	Q1	70	779	11.13	2020.66
3	Plos one	Q2	62	2361	38.08	2013.79
4	International Journal of Molecular Sciences	Q1	52	771	14.83	2019.25
5	Clinical Cancer Research	Q1	47	3614	76.89	2010.94
6	Scientific Reports	Q1	44	791	17.98	2018.25
7	Seminars in Cancer Biology	Q1	44	2689	61.11	2014.68
8	Clinical & Experimental Metastasis	Q2	41	1009	24.61	2011.71
9	Oncogene	Q1	41	2230	54.39	2013.17
10	Oncotarget	-	41	1868	45.56	2015.95

**Table 4:** Top 10 highly co-citation of cited references.

Rank	Year	First Author	Title	Journals	Citations
1	2007	Aguirre-Ghiso, JA	Models, mechanisms and clinical evidence for cancer dormancy	*Nature Reviews Cancer*	428
2	2014	Sosa, MS	Mechanisms of disseminated cancer cell dormancy: an awakening field	*Nature Reviews Cancer*	266
3	1995	HOLMGREN, L	Dormancy of micrometastases-balanced proliferation and apoptosis in the presence of angiogenesis suppression	*Nature Medicine*	245
4	2013	Ghajar, CM	The perivascular niche regulates breast tumor dormancy	*Nature Cell Biology*	236
5	2011	Hanahan, D	Hallmarks of Cancer: The Next Generation	*Cell*	185
6	1998	Luzzi, KJ	Multistep nature of metastatic inefficiency - Dormancy of solitary cells after successful extravasation and limited survival of early micrometastases	*American Journal of Pathology*	183
7	2013	Bragado, P	TGF-beta 2 dictates disseminated tumor cell fate in target organs through TGF-beta-RIII and p38 alpha/beta signaling	*Nature Cell Biology*	166
8	2002	Chambers	Dissemination and growth of cancer cells in metastatic sites	*Nature Reviews Cancer*	164
9	2001	Aguirre-Ghiso, JA	Urokinase receptors and fibronectin regulate the ERK(MAPK) to p38(MAPK) activity ratios that determine carcinoma cell proliferation or dormancy in vivo.	*Molecular Biology of The Cell*	152
10	2003	Aguirre-Ghiso, JA	ERK(MAPK) activity as a determinant of tumor growth and dormancy; regulation by p38(SAPK).	*Cancer Research*	150

**Table 5:** Top 10 key molecules, states and diseases in studies on tumor dormant.

Rank	Molecule	Occurrence	State	Occurrence	Disease	Occurrence
1	Endothelial growth-factor	130	Dormancy	1012	Breast cancer	598
2	Transforming growth factor-beta	79	Metastasis	571	Prostate cancer	192
3	Urokinase receptor	64	Angiogenesis	262	Colorectal cancer	131
4	Growth-factor	53	Epithelial-mesenchymal transition	170	Melanoma	104
5	Nuclear factor-kappa-b	46	Apoptosis	159	Lung cancer	88
6	E-cadherin	43	Proliferation	155	Ovarian cancer	58
7	P53	37	Invasion	82	Hepatocellular carcinoma	39
8	C-myc	35	Self-renewal	76	Pancreatic cancer	31
9	Mcm2–7	32	Autophagy	74	Glioblastoma	21
10	Tumor-necrosis-factor	32	Differentiation	74	Multiple myeloma	20

## Data Availability

The original contributions presented in the study are included in the article; further inquiries can be directed to the corresponding author.

## References

[1] Butturini E., Carcereri de Prati A., Boriero D., Mariotto S. (2019). Tumor Dormancy and Interplay with Hypoxic Tumor Microenvironment. IJMS.

[2] Elkholi I.E., Lalonde A., Park M., Côté J.-F. (2022). Breast Cancer Metastatic Dormancy and Relapse: An Enigma of Microenvironment(s). Cancer Res..

[3] Goss P.E., Chambers A.F. (2010). Does Tumour Dormancy Offer a Therapeutic Target?. Nat. Rev. Cancer.

[4] Santos-de-Frutos K., Djouder N. (2021). When Dormancy Fuels Tumour Relapse. Commun. Biol..

[5] Thompson D.F., Walker C.K. (2015). A Descriptive and Historical Review of Bibliometrics with Applications to Medical Sciences. Pharmacotherapy.

[6] Zielińska A., Karczewski J., Eder P., Kolanowski T., Szalata M., Wielgus K., Szalata M., Kim D., Shin S.R., Słomski R. (2023). Scaffolds for Drug Delivery and Tissue Engineering: The Role of Genetics. J. Control. Release.

[7] Ding X., Yang Z. (2022). Knowledge Mapping of Platform Research: A Visual Analysis Using VOSviewer and CiteSpace. Electron. Commer. Res..

[8] da Silva P.B.V., Brenelli L.B., Mariutti L.R.B. (2023). Waste and By-Products as Sources of Lycopene, Phytoene, and Phytofluene—Integrative Review with Bibliometric Analysis. Food Res. Int..

[9] Hasan M., Abedin M.Z., Amin M.B., Nekmahmud M., Oláh J. (2023). Sustainable Biofuel Economy: A Mapping through Bibliometric Research. J. Env. Manag..

[10] van Eck N.J., Waltman L. (2010). Software Survey: VOSviewer, a Computer Program for Bibliometric Mapping. Scientometrics.

[11] Wang M., Xie X., Zhao S., Han W., Zhang Y. (2022). Global Research Trends and Hotspots of Fecal Microbiota Transplantation: A Bibliometric and Visualization Study. Front. Microbiol..

[12] Settles B. (2005). ABNER: An Open Source Tool for Automatically Tagging Genes, Proteins and Other Entity Names in Text. Bioinformatics.

[13] Maglott D., Ostell J., Pruitt K.D., Tatusova T. (2011). Entrez Gene: Gene-Centered Information at NCBI. Nucleic Acids Res..

[14] von Mering C., Huynen M., Jaeggi D., Schmidt S., Bork P., Snel B. (2003). STRING: A Database of Predicted Functional Associations between Proteins. Nucleic Acids Res..

[15] Shannon P., Markiel A., Ozier O., Baliga N.S., Wang J.T., Ramage D., Amin N., Schwikowski B., Ideker T. (2003). Cytoscape: A Software Environment for Integrated Models of Biomolecular Interaction Networks. Genome Res..

[16] Aguirre-Ghiso J.A. (2021). Translating the Science of Cancer Dormancy to the Clinic. Cancer Res..

[17] Hensel J.A., Flaig T.W., Theodorescu D. (2013). Clinical Opportunities and Challenges in Targeting Tumour Dormancy. Nat. Rev. Clin. Oncol..

[18] Gomatou G., Syrigos N., Vathiotis I.A., Kotteas E.A. (2021). Tumor Dormancy: Implications for Invasion and Metastasis. Int. J. Mol. Sci..

[19] Recasens A., Munoz L. (2019). Targeting Cancer Cell Dormancy. Trends Pharmacol. Sci..

[20] Aguirre-Ghiso J.A. (2007). Models, Mechanisms and Clinical Evidence for Cancer Dormancy. Nat. Rev. Cancer.

[21] Cheung T.H., Rando T.A. (2013). Molecular Regulation of Stem Cell Quiescence. Nat. Rev. Mol. Cell Biol..

[22] Triana-Martínez F., Loza M.I., Domínguez E. (2020). Beyond Tumor Suppression: Senescence in Cancer Stemness and Tumor Dormancy. Cells.

[23] Werner S., Heidrich I., Pantel K. (2022). Clinical Management and Biology of Tumor Dormancy in Breast Cancer. Semin. Cancer Biol..

[24] Humtsoe J.O., Kramer R.H. (2010). Differential Epidermal Growth Factor Receptor Signaling Regulates Anchorage-Independent Growth by Modulation of the PI3K/AKT Pathway. Oncogene.

[25] Schewe D.M., Aguirre-Ghiso J.A. (2008). ATF6alpha-Rheb-MTOR Signaling Promotes Survival of Dormant Tumor Cells in Vivo. Proc. Natl. Acad. Sci. USA.

[26] Bragado P., Estrada Y., Parikh F., Krause S., Capobianco C., Farina H.G., Schewe D.M., Aguirre-Ghiso J.A. (2013). TGF-Β2 Dictates Disseminated Tumour Cell Fate in Target Organs through TGF-β-RIII and P38α/β Signalling. Nat. Cell Biol..

[27] Lu Z., Luo R.Z., Lu Y., Zhang X., Yu Q., Khare S., Kondo S., Kondo Y., Yu Y., Mills G.B. (2008). The Tumor Suppressor Gene ARHI Regulates Autophagy and Tumor Dormancy in Human Ovarian Cancer Cells. J. Clin. Investig..

[28] Balz L.M., Bartkowiak K., Andreas A., Pantel K., Niggemann B., Zänker K.S., Brandt B.H., Dittmar T. (2012). The Interplay of HER2/HER3/PI3K and EGFR/HER2/PLC-Γ1 Signalling in Breast Cancer Cell Migration and Dissemination. J. Pathol..

[29] Sosa M.S., Bragado P., Aguirre-Ghiso J.A. (2014). Mechanisms of Disseminated Cancer Cell Dormancy: An Awakening Field. Nat. Rev. Cancer.

[30] Neophytou C.M., Kyriakou T.-C., Papageorgis P. (2019). Mechanisms of Metastatic Tumor Dormancy and Implications for Cancer Therapy. Int. J. Mol. Sci..

[31] Manjili S.H., Isbell M., Ghochaghi N., Perkinson T., Manjili M.H. (2022). Multifaceted Functions of Chronic Inflammation in Regulating Tumor Dormancy and Relapse. Semin. Cancer Biol..

[32] Cackowski F.C., Heath E.I. (2022). Prostate Cancer Dormancy and Recurrence. Cancer Lett..

[33] Ghajar C.M., Peinado H., Mori H., Matei I.R., Evason K.J., Brazier H., Almeida D., Koller A., Hajjar K.A., Stainier D.Y.R. (2013). The Perivascular Niche Regulates Breast Tumour Dormancy. Nat. Cell Biol..

[34] Mukherjee A., Bravo-Cordero J.J. (2023). Regulation of Dormancy during Tumor Dissemination: The Role of the ECM. Cancer Metastasis Rev..

[35] Tamamouna V., Pavlou E., Neophytou C.M., Papageorgis P., Costeas P. (2022). Regulation of Metastatic Tumor Dormancy and Emerging Opportunities for Therapeutic Intervention. Int. J. Mol. Sci..

[36] Qiu Y., Qiu S., Deng L., Nie L., Gong L., Liao X., Zheng X., Jin K., Li J., Tu X. (2020). Biomaterial 3D Collagen I Gel Culture Model: A Novel Approach to Investigate Tumorigenesis and Dormancy of Bladder Cancer Cells Induced by Tumor Microenvironment. Biomaterials.

[37] Hedley B.D., Chambers A.F. (2009). Tumor Dormancy and Metastasis. Adv. Cancer Res..

[38] Liu Y., Lv J., Liang X., Yin X., Zhang L., Chen D., Jin X., Fiskesund R., Tang K., Ma J. (2018). Fibrin Stiffness Mediates Dormancy of Tumor-Repopulating Cells via a Cdc42-Driven Tet2 Epigenetic Program. Cancer Res..

[39] Manjili M.H. (2017). Tumor Dormancy and Relapse: From a Natural Byproduct of Evolution to a Disease State. Cancer Res..

[40] Nanou A., Lorenzo-Moldero I., Gazouleas K.D., Cortese B., Moroni L. (2022). 3D Culture Modeling of Metastatic Breast Cancer Cells in Additive Manufactured Scaffolds. ACS Appl. Mater. Interfaces.

[41] Kim H., Wirasaputra A., Mohammadi F., Kundu A.N., Esteves J.A.E., Heiser L.M., Meyer A.S., Peyton S.R. (2023). Live Cell Lineage Tracing of Dormant Cancer Cells. Adv. Healthc. Mater..

[42] Adam A.P., George A., Schewe D., Bragado P., Iglesias B.V., Ranganathan A.C., Kourtidis A., Conklin D.S., Aguirre-Ghiso J.A. (2009). Computational Identification of a P38SAPK-Regulated Transcription Factor Network Required for Tumor Cell Quiescence. Cancer Res..

[43] Yu-Lee L.-Y., Yu G., Lee Y.-C., Lin S.-C., Pan J., Pan T., Yu K.-J., Liu B., Creighton C.J., Rodriguez-Canales J. (2018). Osteoblast-Secreted Factors Mediate Dormancy of Metastatic Prostate Cancer in the Bone via Activation of the TGFβRIII–P38MAPK–PS249/T252RB Pathway. Cancer Res..

[44] Ren D., Dai Y., Yang Q., Zhang X., Guo W., Ye L., Huang S., Chen X., Lai Y., Du H. (2019). Wnt5a Induces and Maintains Prostate Cancer Cells Dormancy in Bone. J. Exp. Med..

[45] Capulli M., Hristova D., Valbret Z., Carys K., Arjan R., Maurizi A., Masedu F., Cappariello A., Rucci N., Teti A. (2019). Notch2 Pathway Mediates Breast Cancer Cellular Dormancy and Mobilisation in Bone and Contributes to Haematopoietic Stem Cell Mimicry. Br. J. Cancer.

[46] Attaran S., Bissell M.J. (2022). The Role of Tumor Microenvironment and Exosomes in Dormancy and Relapse. Semin. Cancer Biol..

[47] Globig P., Willumeit-Römer R., Martini F., Mazzoni E., Luthringer-Feyerabend B.J.C. (2022). Slow Degrading Mg-Based Materials Induce Tumor Cell Dormancy on an Osteosarcoma-Fibroblast Coculture Model. Bioact. Mater..

[48] Rossari F., Zucchinetti C., Buda G., Orciuolo E. (2020). Tumor Dormancy as an Alternative Step in the Development of Chemoresistance and Metastasis—Clinical Implications. Cell. Oncol..

[49] Fornetti J., Welm A.L., Stewart S.A. (2018). Understanding the Bone in Cancer Metastasis. J. Bone Min. Res..

[50] Ring A., Spataro M., Wicki A., Aceto N. (2022). Clinical and Biological Aspects of Disseminated Tumor Cells and Dormancy in Breast Cancer. Front. Cell Dev. Biol..

[51] Pranzini E., Raugei G., Taddei M.L. (2022). Metabolic Features of Tumor Dormancy: Possible Therapeutic Strategies. Cancers.

[52] Showalter L., Czerniecki B.J., Koski G.K. (2020). Th1 Cytokines in Conjunction with Pharmacological Akt Inhibition Potentiate Apoptosis of Breast Cancer Cells *in vitro* and Suppress Tumor Growth *in vivo*. Oncotarget.

[53] Bianchini G., Gianni L. (2014). The Immune System and Response to HER2-Targeted Treatment in Breast Cancer. Lancet Oncol..

[54] Correia A.L., Guimaraes J.C., Auf der Maur P., De Silva D., Trefny M.P., Okamoto R., Bruno S., Schmidt A., Mertz K., Volkmann K. (2021). Hepatic Stellate Cells Suppress NK Cell-Sustained Breast Cancer Dormancy. Nature.

[55] Jiang X., Liang L., Chen G., Liu C. (2021). Modulation of Immune Components on Stem Cell and Dormancy in Cancer. Cells.

[56] Chen Y., Gibson S.B. (2021). Three Dimensions of Autophagy in Regulating Tumor Growth: Cell Survival/Death, Cell Proliferation, and Tumor Dormancy. Biochim. Et Biophys. Acta (BBA) Mol. Basis Dis..

[57] Borriello L., Coste A., Traub B., Sharma V.P., Karagiannis G.S., Lin Y., Wang Y., Ye X., Duran C.L., Chen X. (2022). Primary Tumor Associated Macrophages Activate Programs of Invasion and Dormancy in Disseminating Tumor Cells. Nat. Commun..

[58] Zhao L., Zhang K., He H., Yang Y., Li W., Liu T., Li J. (2021). The Relationship Between Mesenchymal Stem Cells and Tumor Dormancy. Front. Cell Dev. Biol..

[59] Stanford-Moore G.B., Canick J., Kaplan S., Lee W.T. (2023). International Collaboration Trends in Facial Plastic and Reconstructive Surgery: A Systematic Bibliometric Scoping Review. JAMA Otolaryngol. Head Neck Surg..

